# Portuguese Honeys from Different Geographical and Botanical Origins: A 4-Year Stability Study Regarding Quality Parameters and Antioxidant Activity

**DOI:** 10.3390/molecules22081338

**Published:** 2017-08-11

**Authors:** Sónia Soares, Diana Pinto, Francisca Rodrigues, Rita C. Alves, M. Beatriz P. P. Oliveira

**Affiliations:** REQUIMTE, LAQV/Department of Chemical Sciences, Faculty of Pharmacy, University of Porto, Rua Jorge Viterbo Ferreira, 228, 4050-313 Porto, Portugal; dianaandreiapinto@gmail.com (D.P.); franciscapintolisboa@gmail.com (F.R.); beatoliv@ff.up.pt (M.B.P.P.O.)

**Keywords:** honey, botanical and geographical origins, colour, antioxidants, storage

## Abstract

Portuguese honeys (*n* = 15) from different botanical and geographical origins were analysed regarding their quality parameters (diastase activity, hydroxymethylfurfural content, moisture and pH), colour (L*, a*, b*) and antioxidant profile (total phenolics content, total flavonoids content, DPPH^•^ scavenging activity, and ferric reducing power). The samples were analysed fresh and after 4-years of storage (at 25 °C and protected from light). The hydroxymethylfurfural content and diastase activity of the fresh samples were in accordance with the recommended values described in the legislation. In general, the antioxidant activity of the samples correlated more with the bioactive compounds content than with colour. The storage affected differently each individual sample, especially regarding the antioxidant profile. Nevertheless, although in general the lightness of the samples decreased (and the redness increased), after 4 years, 11 samples still presented acceptable diastase activity and hydroxymethylfurfural values.

## 1. Introduction

Honey is a natural substance produced by honey bees, being part of the human diet since ancient times. It is produced from the nectar or secretions of living parts of plants, or from the excretions of plant-sucking insects, collected and transformed by bees, deposited, dehydrated and stored in honeycombs [1]. Honey is essentially composed by different sugars (mainly fructose and glucose) and water. In addition, it contains several other minor components such as proteins, free amino acids, minerals, enzymes, vitamins, organic acids and phenolic compounds [2]. Although present in honey in low quantities, these minor compounds are responsible for conferring specific and individual characteristics to honey, depending mainly on botanical and geographical origins, as well as seasonal and environmental factors and the bee species involved in its production [3,4]. Being recognized as a food with healing properties, honey is highly sought after and traditionally used to treat throat disorders, wounds and burns [5]. Thus, several studies were performed to investigate its medicinal properties such as antibacterial and antifungal, gastro and hepatoprotective, antihypertensive, anti-inflammatory, and antioxidant [6,7,8,9,10] and its use for oncological care [11]. 

Although honey is considered a natural and health beneficial product with a high economic value, it can have different commercial value depending on its botanical and geographical origins. Such is the case of monofloral honeys, which arise predominantly from a single botanical origin, being perceived by consumers as a high qualitative honey with distinct individual characteristics [12]. However, previous studies have shown that the quality and biochemical properties of honey are related not only with the nectar source (botanical and geographical origin), but also with climatic conditions, production and processing methods as well as honey maturity [13].

Furthermore, it is expected that some changes could occur in its composition during storage, based on physical and chemical reactions, leading to alterations of honey properties (such as colour or flavour) even when properly processed, packaged and stored [14]. In addition, the impact of storage on some specific honey components (such as antioxidants) is a complex issue, since contradictory results have been reported [15,16]. For instance, Wang et al. reported a decrease of antioxidant activity of honeys after 6 months of storage [15]. In turn, Gheldof and Engeseth, using the same methodology, found no changes of the antioxidant activity of honeys stored for periods longer than 2 years [16]. 

To preserve the safety and health effects, and simultaneously protect consumers, it is of utmost importance to ensure the quality and authenticity of honey. In Portugal, honey production has been increasing, reaching about 12 tons per year, and becoming an important contributor to the national economy [17,18]. However, in the last few years, importation of lower quality cheaper honey have been growing [18,19]. In order to valorise the quality of Portuguese honeys in comparison with the imported ones, several studies were performed regarding physicochemical parameters and biological compounds [9,15,20,21,22,23,24,25,26]. However, none of these studies evaluated the stability of Portuguese honeys over time. In this work, fifteen Portuguese honey samples from distinct botanical and geographical origins, produced in 2012, were evaluated regarding different physicochemical and quality parameters. The same samples were then stored for four years under controlled temperature (25 °C) conditions and a stability study was performed in order to evaluate the influence of storage on the mentioned parameters. 

## 2. Results and Discussion

In this study, honeys of distinct geographical and botanical origins were characterized and compared, fresh and after a 4-year storage period. The production regions included the North and Centre of Portugal, areas highly recognized for their production of high quality honey (Vila Real, Bragança, Chaves, Boticas, Lousã, Penamacor, Guarda, Vila Nova de Foz Côa, and Viseu). Heather, lavender, blueberry, chestnut, orchard, and multifloral were the botanical origins selected. 

Firstly, the quality of all samples were analysed through their HMF content and DA. These parameters are routinely used to evaluate honey freshness, providing information about inadequate processing and/or inappropriate storage conditions. It is expected that a honey of high quality will present a high DA and a low HMF content. According to the current legislation [1,23], DA should be higher than 8 Gothe degrees (except for baker’s honey) and not less than 3 for honeys with low natural enzyme content (e.g., citrus honeys) and, simultaneously, it should present a HMF level lower than 15 mg/kg [23]. In addition, a maximum for HMF content is fixed: 40 mg/kg (except for baker’s honey). For honeys of declared origin from regions with tropical climate and blends of these honeys, the HMF content should be lower than 80 mg/kg.

The DA and HMF contents of the fresh honeys are summarized in Table 1. In general, all samples presented DA and HMF contents in line with the legislation. As can be observed the lowest DA value was obtained for sample 14 (from Bragança) and the highest for sample 6 (from Lousã), with 16.15 and 37.77 Gothe degrees, respectively. In turn, the HMF contents ranged between 0.33 and 17.38 mg/kg, respectively, for sample 9 (from Vila Nova de Foz Côa) and sample 12 (from Penamacor). Generally, the HMF and DA results obtained in the present study are typical of fresh honeys. Moreover, the DA of all samples were higher than 8, suggesting that the honeys were not exposed to high temperatures and were adequately stored after production. However, it is possible to observe a great variation in the HMF content between the honey samples studied. A similar HMF variation have also been reported for other honey samples from different Portuguese regions [19,23,24,25,26]. 

Gomes et al. [19] and Estevinho et al. [24] obtained lower HMF values in honeys from Northeast Portugal and the Trás-os-Montes region (1.14 ± 0.20 mg/kg and 1.1 ± 0.2 mg/kg, respectively) while Pires et al. [27], Feás et al. [28], Estevinho et al. [23] and Iglesias et al. [25] achieved higher HMF results for some samples from different regions of North Portugal. These differences could be attributed to the variation of the several factors that affect HMF formation, such as temperature and time of heating, storage conditions, pH and floral source [29]. 

Moisture values were also assessed (Table 1). The determination of this parameter is related to honey preservation and storage, as high-water content can lead to the growth of molds, causing flavour loss and low shelf-life [25]. The obtained results varied between 13.1 and 16.9, with significant differences (*p* > 0.05) between samples. To guarantee honey safety against fermentation the legislation defined an upper limit of 20% of moisture for honeys. All samples were within the recommended value. However, the differences observed between samples may be due to several factors, such as environmental conditions, harvest period and degree of maturity reached in the hive [30].

In addition, the pH values ranged from 3.98 to 5.05, with significant differences (*p* > 0.05) between samples (Table 1). Although limits for this value are not defined in the legislation, the determination of this parameter is of significant importance as it influences honey texture, stability and shelf-life [31]. Low pH values inhibit the presence and growth of microorganisms and makes honey compatible with many food products in terms of pH and acidity [23]. These moisture and pH values are consistent with previously studies of Portuguese honeys [23,24,25,27,28]. 

Table 2 present the samples colour expressed as mean ± standard deviation of L*, a*, b* parameters. The L* parameter represents the measure of the brightness (lightness) from black (0) to white (100). The a* parameter is the function of the red-green difference, where a positive value indicates red and a negative one represents green (−100/+100). The b* parameter is the function of the green–blue difference. A positive b* value indicates yellow while a negative value represents blue (−100/+100). The units within the L*, a*, b* system give equal perception of the colour difference to a human observer. The results obtained for L* parameter ranged from 33.93 to 47.24 for sample 12 (from Penamacor) and 9 (from Vila Nova de Foz Côa), respectively. In general, the lowest values of L* corresponded to orchard, heather and chestnut honey samples, while lavender and almond blossom honey samples presented the highest values. According to González-Miret et al., honey samples can be classified as light and dark using the L* parameter, where light honeys present high values of L* and dark honeys show the lowest values of this parameter [32]. 

Different authors reported that honey colour is related to TPC, with light-coloured honeys presenting a lower content and the darkest one a high amount [33,34,35,36]. In turn, phenolic compounds were described as the main responsible for the antioxidant activity of honey [7,33,34]. Consequently, darker honeys usually show a higher antioxidant activity than lighter ones [37]. The honey colour, which mainly depends on the nectar source, can also be used as an index of honey’s antioxidant power. 

Table 3 summarizes the results obtained for the TPC and TFC, as well as for the antioxidant activity. Two different assays (DPPH^•^ scavenging activity and FRAP) were selected to assess the antioxidant activity of the samples. These methods were selected since they are simple and relatively fast to carry out, and can give valuable information about the type of antioxidants present in the samples, including about their mechanism of action. In fact, this is possible because these two methods act by two complementary mechanisms of action. The FRAP assay is based on the reduction of ferric 2,4,6-tripyridyl-s-triazine (TPTZ) to a coloured product, detecting several compounds with redox potentials lower than 0.7 V (the redox potential of Fe^3+^-TPTZ), being a reasonable screen for the ability of the samples to maintain the redox status in cells or tissues. In this method, only an electron transfer mechanism occurs [38]. In turn, the DPPH^•^ scavenging assay evaluates the antiradical activity of a sample. The radical can be neutralized either by direct reduction (via electron transfer) or by radical quenching (via H atom transfer) [39]. The lowest TPC was presented by the lavender honey (149.6 mg GAE/kg), while the highest phenolics amount (500.83 mg GAE/kg) was found in the heather honey (sample 5). Indeed, TFC varied from 8.60 to 44.64 mg ECE/kg honey. The lowest values correspond to sample 7 (lavender) and sample 12 (orchard) and the highest TFC belongs to the heather honey from Boticas. A positive correlation was found between TFC and TPC (*r^2^ =* 0.683; *p* < 0.05) for the fresh samples. Other authors also reported a tendency for heather honeys to demonstrate higher phenolic contents [26,40,41]. Gomes et al. [19], Estevinho et al. [24] and Iglesias et al. [25] also evaluated the presence of these compounds in Portuguese honeys, obtaining in general high values when compared with the present study. These compounds are transferred to honey through pollen collected by bees, which can justify the observed variation in TPC and TFC among honey samples. Also, different plants contain different phenolic and flavonoid compounds, presenting fluctuations in their concentration, mainly due to climatic and soil conditions [42]. 

The DPPH^•^ scavenging activity of the prepared extracts ranged from 5.3% (for sample 7) to 48.8% (for sample 11). In what concerns to FRAP assay, the minimum and maximum values were found for sample 7 (497.7 mg FSE/kg) and sample 5 (4086.7 mg FSE/kg), respectively. The highest value was obtained for a heather honey, which in accordance with the results reported by Alves et al. [41] that also described a maximum FRAP value for heather honeys. Based on the results shown in Table 3, it is clear that the antioxidant activity not only depends on the botanical origin of the samples, but also on the geographical origin. In fact, the production of antioxidants is a consequence of plants defense against several environmental factors, including climatic conditions. For instance, it was reported that high-temperature growing conditions (25–30 °C) significantly enhance the antioxidant activity of plants, while those that grown at inferior temperatures (12–18 °C) generally had lower antioxidant activity [43]. Among the conditions that had this effect, ultraviolet radiation, temperature, water stress or mineral nutrient availability were the most important. This can justify that honeys from the same botanical origin, but produced in different regions, present significantly differences in the antioxidant activity. In a previous study, Wilczyńska reported that the highest values of antioxidant activity and TPC were detected in dark honeys (with the lowest L* value) [36]. Similar conclusions were reported by Ferreira et al. [40]. TFC, antioxidant activity and colour were also studied by Kuś et al. [44], reporting a correlation between TFC and colour, as well as with antioxidant activity. Moreover, their results showed a higher TPC in dark buckwheat honey, which also exhibited a higher antioxidant activity. 

In parallel, the in vitro antioxidant capacity observed for honey has also been confirmed by studies that use in vivo models*.* For instance, Ahmad et al. [45] showed that the consumption of Tualang honey (presenting 200.9 mg GAE/kg, a FRAP value of 2555 μmol Fe^2+^/kg and a DPPH^•^ inhibition of 36%) demonstrated a dose-dependent response in increasing antioxidant activity and suppressing oxidative stress in female athletes. The time–course effect that provided optimal antioxidant activity and oxidative stress protection ranged from 1 to 2 h after consumption. Also, Sairazi et al. [46] evaluated the extent of neuroprotective effect conferred by Malaysian Tualang honey in the cerebral cortex of rats against kainic acid (KA), a neurotoxicant extracted from a red algae (*Digenea simplex*). The authors found a therapeutic potential of that honey against KA-induced oxidative stress and neurodegeneration through an antioxidant effect. In another study, Alvarez-Suarez et al. [47] reported that the Manuka honey protected against apoptosis, intracellular reactive oxygen species production, and lipid and protein oxidative damage. Moreover, they also found that the honey protected mitochondrial functionality, promoted cell proliferation and activated the AMPK/Nrf2/ARE (Kelch ECH associating protein 1/NF-E2-related factor 2/antioxidant responsive elements) signalling pathway, as well as the expression of the antioxidant enzymes such as superoxide dismutase and catalase. These properties were attributed to the phenolic compounds present in the sample.

In Table 4, the Pearson correlation coefficients (*r*) between the different parameters analysed in this study are described. Although not very high, positive correlations were found between the L* parameter and TPC (0.383), TFC (0.282), and DPPH^•^ scavenging activity (0.370). In turn, high correlations were observed between TPC and TFC (0.683), TFC and DPPH^•^ scavenging activity (0.646). Finally, an extremely high correlation was observed between TFC and FRAP (0.962), suggesting that, in these samples, TFC has a huge influence in the antioxidant activity when compared to TPC.

The detailed results about the influence of storage on each individual sample can be observed in Table 1, Table 2 and Table 3. In what concerns to DA, a decrease was observed for all samples, albeit still within the allowed legal values. Diastase is an enzyme naturally occurring in honey and its level depends upon geographic and floral origins of the product [25]. However, during storage, diastase can suffer denaturation which contributes to its decrease. 

As expected, an increase in HMF content, ranging from 4.06 to 148.97 mg/kg, was observed, since HMF is produced during acid-catalysed dehydration of hexoses (such as fructose and glucose) and Maillard reactions, which occur in the course of time [30]. 

Moisture and pH were also assessed after the 4-year storage period. In general, moisture values remained stable (ranging between 13.27% and 15.80%) and a slight decrease was observed for pH (with values ranging from 3.58 to 4.50). 

Table 2 summarizes the sample colour results. The lightness decreased significantly (*p* < 0.05) during storage. Simultaneously, there was a tendency of redness (a*) to increase and a general yellowness (b*) decrease (Table 2). These results suggest that honeys become darker during storage, which can be attributed to Maillard reactions, fructose caramelization as well as polyphenol reactions [48].

In what concerns the TPC, an overall increase was detected, except for four honeys (samples 9, 10, 12 and 15). In the same way, TFC also augmented from 2012 to 2016. This is probably due not to a real increase but to the breakdown of phenolic molecules of higher molecular weight as honey ages (a result from enzymatic reactions and/or Maillard reactions) freeing chemical substituent groups with reducing power [14]. These compounds react to a greater extent (compared to the original molecules) with the chemical reagents used in the respective spectrophotometric methods applied. Accordingly, a significant raise (*p* < 0.05) of the reducing power (FRAP) was also noticed. The only exception was sample 15, a multifloral honey, in which a decrease of both TPC (*p* < 0.05) and TFC (not statistically significant) was also observed. The high Pearson coefficient correlations obtained between TPC and FRAP (0.857), as well as for TFC and FRAP (0.796), are indicative that these are the main groups of compounds responsible for the reducing power. Regarding the DPPH^•^ inhibition assay (Table 3), in the majority of the cases, no significant differences (*p* > 0.05) were observed between the results obtained in different years. In two samples, a significant decrease (*p* < 0.05) of the values was observed. The differences obtained between the FRAP and DPPH^•^ inhibition assays suggest that other compounds, besides TPC and TFC, are also contributing to the DPPH^•^ scavenging ability. 

The general data obtained in this study regarding TPC are not in total agreement to those published by Wang et al. [15]. These authors analysed clover and buckwheat honey and reported that after 6 months of storage the TPC decreased. Although in the present study the storage time was longer and a higher number of samples were analysed, a general tendency of TPC increase was observed. However, a decrease of TPC in four of the 15 samples analysed was also detected. In addition, for one sample (sample 8) no significant differences (*p* > 0.05) were found between 2012 and 2016. In addition, Wang et al. reported a significant decrease of antioxidant activity of both honeys (using the ORAC assay) after 6 months of storage [15]. In turn, in another study, Gheldof and Engeseth, using the same methodology, found no variations in the antioxidant activity of honeys for periods longer than 2 years [49]. The results of these authors are more in accordance with the DPPH^•^ inhibition values determined than with the FRAP ones.

**Figure 1:**
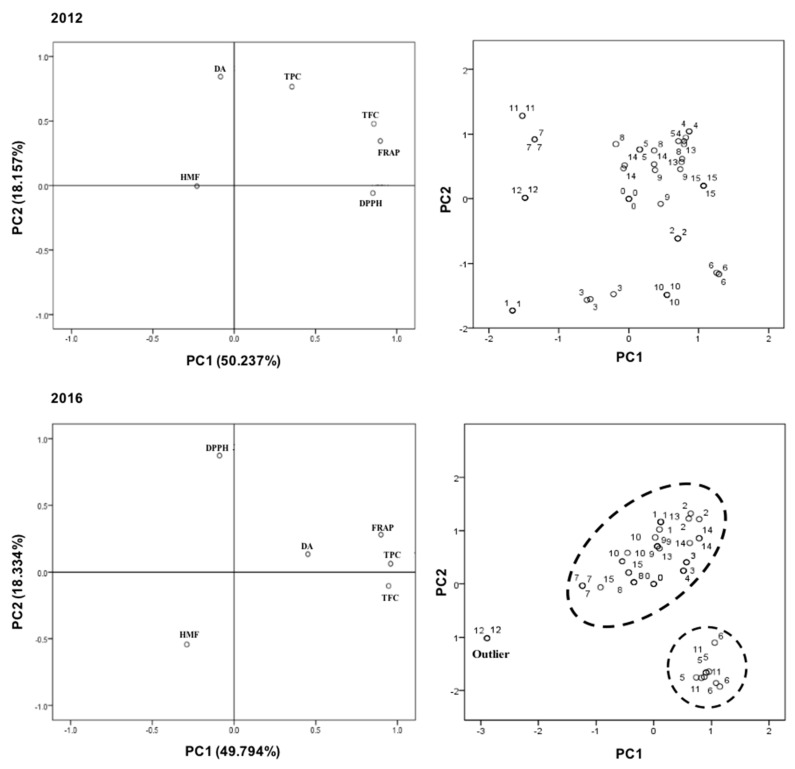
Principal Component Analysis (PCA) - Biplot of scores and loadings of data obtained from HMF, DA, and antioxidant parameters (TPC, TFC, DPPH^•^ SA, FRAP) of the honey samples analysed in 2012 and 2016 (**left**: PCA distribution of variables; **right**: PCA distribution of honey samples).

In order to study the influence of TPC, TFC, DPPH^•^ inhibition, FRAP, colour, HMF and DA on honey quality, a PCA study was carried out. PCA is a recognized technique that allows the evaluation of the influence of several factors on a final value. The objective of a loading projection is to visualize the position of the variables with respect to one another in two-dimensional space and their corresponding correlations. Variables closest and far from the plot origin are positively correlated while variables opposite one another on the plot are negatively correlated. In what concerns to the fresh samples (analysed in 2012) (Figure 1), the two first principal components (PC1 and PC2) explained 50.273% and 18.157% of the variability between samples, respectively. In the PCA analysis of 2012, PC1 is mainly related with antioxidant activity and TFC and PC2 is positively associated with DA. It can also be observed that the fresh samples presented a higher heterogeneity compared to the stored samples (2016). 

In fact, after being subjected to a 4-year storage period at 25 °C, some modifications were detected in the samples. TPC, TFC and FRAP values obtained in 2016 are closely related with PC1 while DPPH^•^ inhibition is strongly related with PC2. The first principal component had the highest eigenvalue of 2.988, and accounted for 49.794% of the variability in the data set while PC2 is responsible for 18.334% and presented an eigenvalue of 1.1. An overall view using the PCA shows that, in a general way, the TPC and HMF values were higher in 2016 than in 2012. Moreover, the PCA correlation plots show the clustering of the stored honey samples into two main groups, revealing an increase of the samples homogeneity (Figure 1). In general, the differences obtained among the samples for all the antioxidant parameters studied (TPC, TFC, DPPH^•^ scavenging activity, and FRAP) clearly show that the storage conditions affect distinct types of honey in a different way, being the results mainly dependent on the original honey composition.

## 3. Materials and Methods

### 3.1. Standards and Reagents

Ascorbic acid, 1,1-diphenyl-2-picrylhydrazyl (DPPH^•^) free radical, epicatechin, Folin–Ciocalteu’s reagent, gallic acid, and iodine were purchased from Sigma-Aldrich (Steinheim, Germany). Methanol (reagent grade), sodium acetate, sodium carbonate decahydrate, sodium nitrite, aluminium chloride and sodium hydroxide were purchased from Merck (Darmstadt, Germany). All other reagents were of analytical grade.

### 3.2. Samples

**Figure 2:**
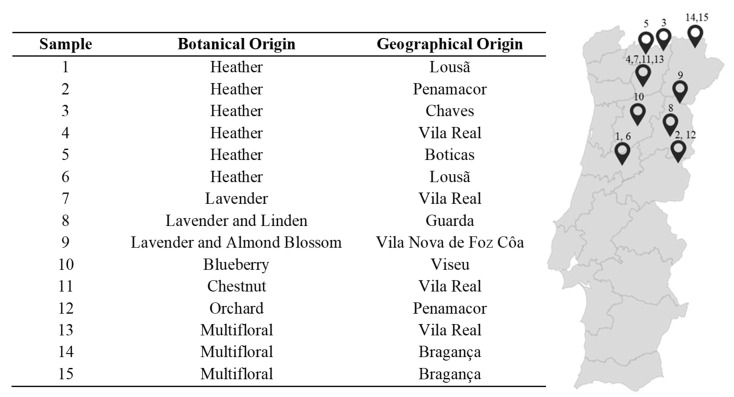
Description of the Portuguese honey samples analysed in this study regarding their botanical and geographical origins.

Fifteen honey samples, from distinct botanical origins and produced in 2012, were acquired in the North and Centre of Portugal. Samples were acquired in labelled glass containers, with at least 500 g of honey, directly from producers. A detailed description of the samples is presented in Figure 2. After purchase, samples were immediately analysed, according to the following sections. The influence of storage time on the studied properties were evaluated after 4 years (in 2016). This period was selected considering the maximum storage validity mentioned on the labels of the analysed samples. During this period, samples were stored in the original package at controlled temperature (25 °C) in the dark. This temperature was selected considering the normal storage conditions mentioned on the labels.

### 3.3. Colour Analysis

Measurements were performed using a colorimeter (Chroma Meter CR 410, Konica Minolta, Tokyo, Japan), previously calibrated with a white standard plate. Samples were placed on a Petri plate with 5.5 cm diameter, with a sample thickness of 5 mm. Colour indices (a*, b* and L*) and the hue angle (h*), chroma (C*) and total colour difference (ΔE*) were calculated as:

(1) $$h^{*}=\tan^{-1}\left(\frac{b^{*}}{a^{*}}\right)$$

(2) $$C^{*}={\left[{\left(a^{*}\right)}^{2}+{\left(b^{*}\right)}^{2}\right]}^{1/2}$$

(3) $$∆E^{*}={\left[{\left(∆L^{*}\right)}^{2}+{\left(∆a^{*}\right)}^{2}+{\left(∆b^{*}\right)}^{2}\right]}^{1/2}$$

Colour measurements were performed in triplicate. 

### 3.4. pH

The pH of the honey samples was directly analysed with a pH meter (Crison Instruments, Barcelona, Spain) equipped with a glass electrode. The measurements were performed in triplicate.

### 3.5. Moisture

The moisture content was determined using a refractometer (readings were performed at 20 °C). The corresponding moisture values (%) were obtained from a reference Chataway Table [50]. The analyses were performed in triplicate.

### 3.6. Diastase Activity (DA)

DA was determined according to the AOAC method 958.09 [51]. An amount of honey (10 g) was rigorously weighed into a 50-mL beaker and dissolved in water (20 mL) and acetate buffer solution (5 mL, pH = 5.3). The mixture was transferred to a 50-mL volumetric flask containing a NaCl solution (3 mL, 0.5 M) and the final volume was completed with water. A tube containing 10 mL of this solution was placed into a water bath at 40 °C along with another tube containing a 2% starch solution. After 15 min, starch solution (5 mL) was carefully dispensed into the reaction tube with the honey solution. Contents were mixed and timed. An aliquot (1 mL) was removed at 5 min intervals and placed in beakers with 10 mL of diluted iodine solution (3.5 × 10^−4^ M) and a known amount of deionised water (equal to the volume necessary for the standardization of the starch solution). The absorbance of this mixture was immediately measured at 660 nm against a water blank in a UV-1800 spectrophotometer (Shimadzu, Kyoto, Japan). A plot of absorbance against time was used to determine the time, *tx*, at which the specific absorbance of 0.235 was reached. The diastase index (DI) was calculated using the equation: DI = 300/*tx* and the results were expressed in Gothe degrees. This unit is defined as the amount of enzyme which hydrolyses 0.01 g of starch for one hour at 40 °C, under standard conditions.

### 3.7. Hydroxymethylfurfural Quantification

An aliquot of honey (5 g) was rigorously weighted and mixed with deionised water (50.0 mL). The mixture was filtered through a PTFE membrane (0.22 µm) before HPLC analysis. The integrated HPLC system used (Jasco, Tokyo, Japan) was composed by an AS-950 automated injector, a PU-980 pump, and a MD-2010 Plus multiwavelength diode-array detector (DAD). The chromatographic separation was achieved with a RP-Tracer Excel ODS-A column (5 µm; 250 mm × 4 mm), from Teknokroma (Barcelona, Spain), using an isocratic solvent system of water: acetonitrile (80:20, *v*/*v*). Elution was performed at a solvent flow rate of 1 mL/min. Chromatograms were recorded at 285 nm. Chromatographic data was analysed using a Borwin-PDA Controller Software (JMBS, Le Fontanil, France). 

### 3.8. Antioxidant Activity Analysis

#### 3.8.1. DPPH^•^ Scavenging Activity

The antiradical activity of the samples against DPPH^•^ was evaluated according to Alves et al. [20] with minor modifications to adapt volumes to a microplate reader. Briefly, a sample (30 µL) diluted in methanol was added to a DPPH^•^ solution (270 µL, 6 × 10^−5^ M). The absorbance decrease was followed at 517 nm until a stable value was achieved (Sinergy^TM^ HT, Biotek Instruments, Winooski, VT, USA). Analyses were performed in triplicate and the results expressed as percentage of scavenging activity (% DPPH^•^ SA), which was calculated according to the following equation:

% DPPH^•^ SA = [(Abs_it_ − Abs_ft_)/Abs_it_] × 100, (4)

where Abs_it_ is the absorbance of the DPPH^•^ solution at the initial time of reaction (0 min) and Abs_ft_ is the absorbance of the DPPH^•^ solution after reaction with the sample extract (final time). Analyses were performed in triplicate.

#### 3.8.2. Ferric Reducing Antioxidant Power Assay

The reducing power of the honey samples was analysed according to Costa et al. with minor modifications [21]. Briefly, 90 µL of a sample dilution in methanol, 270 µL of distilled water and 2.7 µL of FRAP reagent (containing 0.3 M acetate buffer, 10 mM TPTZ-solution, and 20 mM ferric chloride) was mixed and incubated in a water bath at 37 °C, during 15 min. Absorbance was measured at 595 nm (Sinergy^TM^ HT). A calibration curve was prepared using ferrous sulphate (0–2000 μM, R^2^ = 0.9982). FRAP values were expressed as mg of ferrous sulphate equivalents (FSE) per kg of honey. Analyses were performed in triplicate.

### 3.9. Total Phenolics Content

Total phenolics content (TPC) was determined according to Alves et al. with minor modifications [20]. Briefly, a sample (500 µL) diluted with methanol was mixed with Folin–Ciocalteu reagent (2.5 mL, 1:10) and a Na_2_CO_3_^•^10 H_2_O solution (2 mL, 7.5%). The mixture was incubated, protected from light, at 45 °C during 15 min. Afterwards, it was placed at room temperature, in the absence of light, during 30 min. The absorbance measurements were performed at 765 nm against a distilled water blank (Synergy^TM^ HT). A calibration curve was prepared using gallic acid as standard (0–100 mg/L, R^2^ = 0.9991). The TPC of samples was expressed as mg of gallic acid equivalents (GAE) per kg of honey. Analyses were performed in triplicate.

### 3.10. Total Flavonoids Content

Total flavonoids content (TFC) was determined according to Costa et al. with minor modifications [21]. Briefly, distilled water (4 mL) and NaNO_2_ solution (300 µL, 5 g/100 mL) was added to a sample diluted with methanol (1 mL). After 5 min, 10% (*w*/*v*) AlCl_3_ solution (300 µL) were added, and after 1 min, 1 M NaOH (2 mL) and deionised water (2.4 mL) were also added. The absorbance measurements were carried out at 510 nm against a blank (Synergy^TM^ HT). Epicatechin was used as standard to prepare the calibration curve (0–100 mg/L, R^2^ = 0.9999). TFC was expressed as mg of epicatechin equivalents (ECE) per kg of honey. Analyses were performed in triplicate.

### 3.11. Statistical Analysis

Data were reported as mean ± standard deviation of at least triplicate experiments. Statistical analysis of the results was performed with the software SPSS 24.0 (SPSS Inc., Chicago, IL, USA). One-way ANOVA was used to investigate the differences between samples. Post-hoc comparisons were performed according to Tukey’s HSD test. In all cases, *p* < 0.05 was accepted as significant. Correlations among the different parameters analysed were achieved by Pearson correlation coefficients (*r*) at a significance level of 95% (*p* < 0.05). Principal components analysis (PCA) was applied as pattern recognition unsupervised classification method. The number of dimensions to keep for data analysis was assessed by the respective eigenvalues (which should be greater than one), by the Cronbach’s alpha parameter (that must be positive) and also by the total percentage of variance (that should be as high as possible) explained by the number of components selected. The number of plotted dimensions was chosen in order to allow meaningful interpretations.

## 4. Conclusions

The honey samples analysed in this study were characterized regarding their quality parameters (DA, HMF, moisture and pH), colour and antioxidant profile (TPC, TFC, DPPH^•^ scavenging activity). The HMF and DA of the fresh samples were in accordance with the recommended values described in the legislation. Significant differences were found between samples due to their distinct botanical and geographical origins, but in general, the antioxidant activity was more correlated with the bioactive compounds content than with colour. The 4-years of storage affected each individual sample differently, and after the 4 years 11 samples still presented very acceptable values of DA and HMF. Nevertheless, in general, sample lightness decreased and redness increased. Also, a tendency of TPC and TFC to increase was noticed. The effect of storage on antioxidant activity varied not only with the sample, but also with the methodology employed.

## Figures and Tables

**Table 1:** Physicochemical parameters of honey samples in 2012 and 2016 (mean ± standard deviation, *n* = 3).

Sample	HMF (mg/kg)		DA (Gothe Degrees)		pH		Moisture (%)	
	2012	2016	2012	2016	2012	2016	2012	2016
1	16.99 ± 0.52 ^a^	55.11 ± 0.87 ^d,^*	19.90 ± 0.58 ^g,h,i^	15.83 ± 0.29 ^c,d,e,f,^*	4.52 ± 0.04 ^f^	3.92 ± 0.01 ^h,^*	14.04 ± 0.14 ^c,d,e,f,g^	14.00 ± 0.00 ^f,g^
2	2.38 ± 0.25 ^c^	12.89 ± 0.12 ^g,h,^*	27.78 ± 0.76 ^c^	19.23 ± 0.11 ^a,^*	4.88 ± 0.00 ^b^	4.50 ± 0.01 ^a,^*	13.14 ± 0.01 ^g^	13.47 ± 0.09 ^h,i,^*
3	1.13 ± 0.06 ^c,d,e,f^	20.99 ± 0.07 ^e,f,^*	32.64 ± 0.85 ^b^	17.83 ± 0.22 ^a,b,c,^*	4.80 ± 0.00 ^c,d^	4.26 ± 0.01 ^d,^*	13.77 ± 0.02 ^c,e,f,g^	14.40 ± 0.00 ^d,e,^*
4	2.22 ± 0.03 ^c,d^	4.15 ± 0.06 ^i,^*	24.80 ± 0.45 ^d,e^	15.68 ± 0.18 ^d,e,f,g,^*	5.05 ± 0.01 ^a^	4.21 ± 0.00 ^e,^*	16.85 ± 0.09 ^d,e,f,g^	15.67 ± 0.12 ^b,^*
5	1.73 ± 0.08 ^c,d^	23.31 ± 0.00 ^e,^*	21.74 ± 1.06 ^f,g^	15.57 ± 0.38 ^e,f,g,^*	4.74 ± 0.01 ^d^	4.37 ± 0.01 ^b,^*	13.97 ± 0.21 ^b,c,d,e^	15.00 ± 0.00 ^i,^*
6	7.70 ± 0.18 ^b^	105.05 ± 2.14 ^c,^*	37.77 ± 1.41 ^a^	18.10 ± 0.19 ^a,b,^*	4.82 ± 0.00 ^b,c^	4.30 ± 0.01 ^c,^*	15.90 ± 0.41 ^e,f,g^	14.40 ± 0.00 ^e,f,^*
7	0.71 ± 0.06 ^e,f^	15.21 ± 0.31 ^f,g,^*	20.85 ± 1.22 ^g,h^	8.88 ± 0.58 ^h,^*	3.98 ± 0.05 ^i^	3.80 ± 0.01 ^i,^*	15.40 ± 0.20 ^a,b^	14.80 ± 0.00 ^d,e,^*
8	0.79 ± 0.03 ^e,f^	21.00 ± 0.22 ^e,f,^*	21.68 ± 0.93 ^f,g^	17.69 ± 0.27 ^a,b,c,d,^*	4.60 ± 0.01 ^e^	3.91 ± 0.01 ^h,^*	15.16 ± 0.94 ^c,d,e,f,g^	13.27 ± 0.12 ^g,h^
9	0.33 ± 0.01 ^f^	22.49 ± 0.13 ^e,^*	18.93 ± 1.18 ^i^	17.69 ± 0.27 ^a,b,c,d^	4.64 ± 0.02 ^e^	4.11 ± 0.01 ^f,^*	15.37 ± 0.79 ^f,g^	14.20 ± 0.20 ^h,i^
10	7.11 ± 0.25 ^b^	115.95 ± 0.01 ^b,^*	26.48 ± 0.80 ^c,d^	17.39 ± 0.21 ^a,b,c,d,e,^*	4.34 ± 001 ^g^	3.96 ± 0.01 ^g,^*	14.73 ± 0.06 ^b,c,d^	14.67 ± 0.23 ^e,f^
11	1.06 ± 0.17 ^d,e,f^	4.06 ± 0.04 ^i,^*	19.79 ± 0.47 ^h,i^	16.93 ± 0.31 ^b,c,d,e,f,^*	5.01 ± 0.01 ^a^	4.26 ± 0.01 ^d,^*	14.29 ± 0.40 ^b,c,d,e,f^	13.73 ± 0.12 ^c,d^
12	17.38 ± 1.05 ^a^	148.97 ± 6.14 ^a,^*	19.62 ± 0.33 ^h,i^	13.60 ± 0.43 ^g,^*	4.01 ± 0.03 ^i^	3.58 ± 0.01 ^j,^*	13.65 ± 0.89 ^a^	13.47 ± 0.12 ^a^
13	1.01 ± 0.00 ^d,e,f^	14.14 ± 0.11 ^g,h,^*	25.32 ± 0.89 ^d^	17.48 ± 1.70 ^a,b,c,d,e,^*	4.49 ± 0.00 ^f^	4.36 ± 0.02 ^b,^*	13.91 ± 0.76 ^b^	14.20 ± 0.00 ^b,c^
14	0.74 ± 0.03 ^e,f^	15.31 ± 0.03 ^f,g,^*	16.15 ± 0.13 ^j^	15.68 ± 0.37 ^d,e,f,g^	4.09 ± 0.01 ^h^	4.12 ± 0.01 ^f,^*	14.68 ± 0.12 ^b,c,d,e,f^	14.40 ± 0.00 ^d,e^
15	0.81 ± 0.04 ^e,f^	7.65 ± 0.04 ^h,i,^*	23.40 ± 0.24 ^e,f^	15.00 ± 0.41 ^f,g,^*	4.60 ± 0.01 ^e^	3.61 ± 0.02 ^j,^*	14.57 ± 0.0 ^b,c,d,e,f^	15.80 ± 0.00 ^a,^*

Different letters in the same column indicate significant differences (*p* < 0.05) between samples; * means a significant difference (*p* < 0.05) between the results obtained in 2012 and 2016 for the same sample. HMF, hydroxymethylfurfural; DA, diastase activity.

**Table 2:** Color variation during honey storage between 2012 and 2016.

Sample	2012			2016		
	L* (Lightness)	a* (Redness)	b* (Yellowness)	L* (Lightness)	a* (Redness)	b* (Yellowness)
1	37.71 ± 0.03 ^e,f^	3.52 ± 0.01 ^a^	10.06 ± 0.02 ^g^	17.74 ± 0.35 ^d,^*	2.77 ± 0.11 ^d^	1.85 ± 0.07 ^g,^*
2	39.51 ± 0.00 ^d^	2.73 ± 0.03 ^b^	12.52 ± 0.01 ^e^	18.68 ± 0.35 ^c,d,^*	3.45 ± 0.08 ^c,^*	2.84 ± 0.06 ^f,^*
3	38.67 ± 0.00 ^d,e^	−2.06 ± 0.02 ^i^	8.42 ± 0.01 ^h^	17.90 ± 0.16 ^d,^*	3.28 ± 0.02 ^c,^*	2.04 ± 0.03 ^g,^*
4	37.91 ± 0.19 ^e,f^	−0.28 ± 0.07 ^f^	8.16 ± 0.14 ^h^	21.26 ± 0.40 ^b,^*	4.32 ± 0.13 ^b,^*	4.25 ± 0.06 ^d,^*
5	37.09 ± 0.51 ^f^	2.24 ± 0.14 ^c^	7.36 ± 0.08 ^i^	17.09 ± 0.25*	0.98 ± 0.01 ^f^	0.73 ± 0.04 ^h,^*
6	41.68 ± 0.04 ^c^	0.88 ± 0.01 ^e^	14.96 ± 0.04 ^c^	20.02 ± 0.45 ^b,c,^*	4.68 ± 0.20 ^a,b,^*	3.52 ± 0.11 ^e,^*
7	44.58 ± 0.03 ^b^	−1.92 ± 0.02 ^i^	17.81 ± 0.01 ^a^	21.53 ± 0.43 ^a,b,^*	1.80 ± 0.09 ^e,^*	5.57 ± 0.16 ^b,^*
8	41.47 ± 0.04 ^c^	1.11 ± 0.03 ^e^	14.98 ± 0.00 ^c^	23.02 ± 0.25 ^a,^*	3.61 ± 0.16 ^c,^*	6.60 ± 0.22 ^a^*
9	47.24 ± 0.13 ^a^	−4.26 ± 0.03 ^k^	18.42 ± 0.11 ^a^	20.63 ± 0.13 ^b,^*	4.44 ± 0.04 ^b^*	4.77 ± 0.03 ^c,^*
10	41.16 ± 0.01 ^c^	1.67 ± 0.00 ^d^	13.56 ± 0.05 ^d^	21.42 ± 0.25 ^a,b,^*	4.95 ± 0.20 ^a,^*	5.62 ± 0.18 ^b,^*
11	36.70 ± 0.14	−1.38 ± 0.00 ^h^	6.91 ± 0.03 ^i^	17.74 ± 0.71 ^d,^*	0.93 ± 0.07 ^f,^*	0.95 ± 0.04 ^h,^*
12	33.93 ± 0.21 ^g^	−0.73 ± 0.02 ^g^	4.09 ± 0.05 ^j^	20.25 ± 0.30 ^b,c,^*	1.95 ± 0.13 ^e,^*	2.80 ± 0.18 ^f,^*
13	39.67 ± 0.71 ^d^	−0.25 ± 0.13 ^f^	10.82 ± 0.37 ^f^	21.34 ± 0.95 ^b,^*	3.46 ± 0.05 ^c,^*	5.81 ± 0.19 ^b^
14	45.08 ± 0.15 ^b^	−3.60 ± 0.01 ^j^	15.95 ± 0.32 ^b^	18.25 ± 0.05 ^d,^*	1.07 ± 0.05 ^f,^*	1.64 ± 0.05 ^g,^*
15	38.21 ± 0.26 ^e^	3.50 ± 0.23 ^a^	10.69 ± 0.50 ^f,g^	18.64 ± 0.31 ^c,d,^*	1.06 ± 0.07 ^f,^*	2.03 ± 0.03 ^g,^*

Different letters in the same column indicate significant differences (*p* < 0.05) between samples; * means a significant difference (*p* < 0.05) between the results obtained in 2012 and 2016 for the same sample.

**Table 3:** Descriptive statistics of storage effect on antioxidant activity and related compounds of the honeys studied.

Sample	TPC (mg GAE/kg)		TFC (mg ECE/kg)		DPPH^•^ SA (%)		FRAP (mg FSE/kg)	
	2012	2016	2012	2016	2012	2016	2012	2016
1	269.03 ± 2.01 ^f^	301.70 ± 1.95 ^f,^*	21.77 ± 0.92 ^d,e^	48.58 ± 2.52 ^c,d,^*	24.6 ± 0.2 ^d^	21.2 ± 0.4 ^c,d^	1948.0 ± 24.2 ^a,b,c^	2170.8 ± 4.2 ^b,c^*
2	327.34 ± 4.02 ^d^	380.40 ± 8.92 ^d,^*	30.08 ± 0.89 ^c^	54.83 ± 9.53 ^c,d^	18.5 ± 0.4 ^e^	16.2 ± 0.9 ^f,g^	2956.0 ± 20.7 ^a,b,c^	3282.7 ± 7.3 ^a,b,c^*
3	300.32 ± 12.56 ^e^	382.79 ± 8.49 ^d,^*	23.15 ± 0.02 ^d^	49.47 ± 2.19 ^c,d,^*	18.2 ± 0.3 ^e^	16.4 ± 0.3 ^f,g^	2225.7 ± 9.8 ^a,b,c^	2957.7 ± 4.2 ^a,b,c,^*
4	251.97 ± 2.01 ^g^	335.09 ± 1.95 ^e,^*	17.61 ± 1.96 ^f^	36.98 ± 1.26 ^d,e,^*	27.9 ± 0.5 ^c^	20.5 ± 0.6 ^c,d,e^	1728.3 ± 16.5 ^a,c^	3062.7 ± 7.3 ^b,c^*
5	500.83 ± 3.48 ^a^	591.87 ± 11.74 ^a^	44.64 ± 0.95 ^a^	111.04 ± 2.19 ^a,b,^*	33.2 ± 0.1 ^b^	31.0 ± 1.3 ^b^	4086.7 ± 74.3 ^b,c^	4863.8 ± 16.9 ^a,b,c,^*
6	378.53 ± 4.02 ^c^	461.49 ± 1.95 ^c,^*	31.47 ± 0.03 ^c^	94.09 ± 12.81 ^b,^*	33.3 ± 0.2 ^b^	30.1 ± 0.9 ^b^	2286.7 ± 36.4 ^a,b,c^	3067.6 ± 8.5 ^b,c,^*
7	149.58 ± 2.01 ^l^	267.51 ± 10.73 ^g,^*	8.60 ± 0.02 ^i^	17.35 ± 5.78 ^f^	5.3 ± 0.1 ^g^	7.0 ± 1.2 ^h^	497.7 ± 21.1 ^c^	1598.9 ± 16.9 ^c,^*
8	220.68 ± 2.01 ^h,i^	232.53 ± 8.49 ^h^	14.15 ± 0.95 ^g^	26.27 ± 1.26 ^e,f,^*	32.5 ± 0.2 ^b^	22.6 ± 0.7 ^c,^*	989.3 ± 9.8 ^a,b,c^	1708.9 ± 18.5 ^a,b^*
9	378.53 ± 4.02 ^c^	297.72 ± 7.37 ^f,^*	19.69 ± 0.88 ^e,f^	53.93 ± 2.52 ^c,d,^*	13.0 ± 0.9 ^f^	15.0 ± 0.3 ^g^	1706.3 ± 9.8 ^a^	2109.7 ± 19.4 ^a,b^*
10	395.60 ± 2.01 ^b^	280.23 ± 3.89 ^f,g,^*	23.85 ± 1.01 ^d^	43.23 ± 2.52 ^d,e,^*	18.9 ± 0.2 ^e^	19.0 ± 0.7 ^d,e,f^	1631.3 ± 17.2 ^c^	2139.0 ± 14.7 ^c,^*
11	183.71 ± 2.01 ^k^	509.19 ± 11.85 ^b,^*	37.01 ± 0.99 ^b^	112.83 ± 5.50 ^a,^*	48.8 ± 0.6 ^a^	47.5 ± 0.3 ^a^	3944.7 ± 38.2 ^a,b,c^	5130.2 ± 12.7 ^a,b,c,^*
12	200.77 ± 2.01 ^j^	139.52 ± 10.84 ^i,^*	10.68 ± 0.01 ^h^	16.46 ± 2.52 ^f^	18.7 ± 0.5 ^e^	17.3 ± 0.4 ^e,f,g,^*	764.7 ± 4.6 ^a,b,c^	1391.2 ± 7.3 ^a,b,c^*
13	224.95 ± 4.02 ^h^	293.75 ± 4.90 ^f,g,^*	17.61 ± 0.87 ^f^	28.06 ± 0.00 ^e,f,^*	11.9 ± 0.0 ^f^	9.7 ± 1.7 ^h^	1506.0 ± 5.2 ^a,b,c^	3827.6 ± 18.5 ^a,b,c,^*
14	207.88 ± 4.02 ^i,j^	389.94 ± 3.89 ^d,^*	14.15 ± 0.92 ^g^	63.75 ± 1.26 ^c,^*	18.6 ± 0.8 ^e^	16.9 ± 0.4 ^f,g^	914.3 ± 9.8 ^a,b,c^	3162.9 ± 23.6 ^a,b,c,^*
15	378.53 ± 2.01 ^c^	288.98 ± 5.95 ^f,g,^*	31.47 ± 0.03 ^c^	25.38 ± 4.37 ^e,f^	27.0 ± 0.6 ^c^	31.0 ± 1.3 ^b^	2656.3 ± 9.8 ^a,b^	1540.3 ± 15.3 ^a,^*

Data are expressed as mean ± standard deviation (*n* = 3). Different letters in the same column indicate significant differences (*p* < 0.05) between samples for a certain parameter; * means a significant difference (*p* < 0.05) between the results obtained in 2012 and 2016 for the same sample. TPC, total phenolics content; GAE, gallic acid equivalents; TFC, total flavonoid contents; ECE, epicatechin equivalents; DPPH^•^ SA, DPPH^•^ scavenging activity; FRAP, ferric reducing antioxidant power; FSE, ferrous sulphate equivalents.

**Table 4:** Pearson correlation coefficients between TPC, TFC, DPPH^•^ SA, FRAP, color parameters, HMF and DA for honey samples analysed in 2012 and 2016, expressed as *p*-levels (95% confidence limit).

2012									
	TPC	TFC	DPPH^•^ SA	FRAP	L*	a*	b*	HMF	DA
TPC	-	0.683	0.143	0.546	0.383	0.395	0.322	−0.027	0.149
TFC	0.683	-	0.646	0.962	0.282	−0.222	0.244	−0.194	−0.167
DPPH^•^ SA	0.143	0.646	-	0.657	0.370	−0.095	0.372	−0.191	−0.400
FRAP	0.546	0.962	0.657	-	0.185	−0.163	0.150	−0.180	−0.241
*L*	0.383	0.282	0.370	0.185	-	−0.517	0.955	−0.463	0.062
*a*	0.395	−0.222	−0.095	−0.163	−0.517	-	−0.293	0.330	0.084
*b*	0.322	0.244	0.372	0.150	0.955	−0.293	-	−0.379	0.104
HMF	−0.027	−0.194	−0.191	−0.180	−0.463	0.330	−0.379	-	−0.285
DA	0.149	−0.167	−0.400	−0.241	0.062	0.084	0.104	−0.285	-
**2016**									
	TPC	TFC	DPPH^•^ SA	FRAP	L*	a*	b*	HMF	DA
TPC	-	0.911	0.596	0.857	−0.043	0.022	−0.039	0.371	−0.096
TFC	0.911	-	0.685	0.796	−0.095	−0.081	−0.093	−0.206	0.194
DPPH^•^ SA	0.596	0.685	-	0.496	0.061	−0.134	0.039	−0.255	−0.042
FRAP	0.857	0.796	0.496	-	−0.095	−0.216	−0.116	−0.335	0.275
*L*	−0.043	−0.095	0.061	−0.095	-	0.569	0.947	0.174	−0.123
*a*	0.022	−0.081	−0.134	−0.216	0.569	-	0.643	0.492	0.291
*b*	−0.039	−0.093	0.039	−0.116	0.947	0.643	-	−0.036	0.095
HMF	0.371	−0.206	−0.255	−0.335	0.174	0.492	−0.036	-	−0.026
DA	−0.096	0.194	−0.042	0.275	−0.123	0.291	0.095	−0.026	-

TPC, total phenolics content; TFC, total flavonoid contents; DPPH^•^ SA, DPPH^•^ scavenging activity; FRAP, ferric reducing antioxidant power; HMF, hydroxymethylfurfural; DA, diastase activity.
